# Spontaneous migraine attack causes alterations in default mode network connectivity: a resting-state fMRI case report

**DOI:** 10.1186/s13104-017-2484-1

**Published:** 2017-04-26

**Authors:** Andrea Edit Edes, Lajos Rudolf Kozak, Mate Magyar, Terezia Zsombok, Gyongyi Kokonyei, Gyorgy Bagdy, Gabriella Juhasz

**Affiliations:** 1 MTA-SE-NAP B Genetic Brain Imaging Migraine Research Group, Hungarian Academy of Sciences, Semmelweis University, Nagyvárad Square 4, Budapest, H-1089 Hungary; 2 MTA-SE Neuropsychopharmacology and Neurochemistry Research Group, Hungarian Academy of Sciences, Semmelweis University, Nagyvárad Square 4, Budapest, H-1089 Hungary; 3 Department of Pharmacodynamics, Faculty of Pharmacy, Semmelweis University, Nagyvárad Square 4, Budapest, H-1089 Hungary; 4 MR Research Center, Semmelweis University, Balassa Street 6, Budapest, H-1083 Hungary; 5 Department of Neurology, Faculty of Medicine, Semmelweis University, Balassa Street 6, Budapest, H-1083 Hungary; 6 Institute of Psychology, Eotvos Lorand University, Izabella Street 46, Budapest, H-1064 Hungary; 7 NAP-A-SE Research Group, Hungarian Academy of Sciences, Semmelweis University, Nagyvárad Square 4, Budapest, H-1089 Hungary; 8 Neuroscience and Psychiatry Unit, Manchester Academic Health Sciences Centre, The University of Manchester, Stopford Building, Oxford Road, M13 9PT, Manchester, UK

**Keywords:** Neuroimaging, Functional connectivity, Migraine, Migraine attack, Headache pain, Pain processing, Case report

## Abstract

**Background:**

Although migraine is one of the most investigated neurologic disorders, we do not have a perfect neuroimaging biomarker for its pathophysiology. One option to improve our knowledge is to study resting-state functional connectivity in and out of headache pain. However, our understanding of the functional connectivity changes during spontaneous migraine attack is partial and incomplete.

**Case presentation:**

Using resting-state functional magnetic resonance imaging we assessed a 24-year old woman affected by migraine without aura at two different times: during a spontaneous migraine attack and in interictal phase. Seed-to-voxel whole brain analysis was carried out using the posterior cingulate cortex as a seed, representing the default mode network (DMN). Our results showed decreased intrinsic connectivity within core regions of the DMN with an exception of a subsystem including the dorsal medial and superior frontal gyri, and the mid-temporal gyrus which is responsible for pain interpretation and control. In addition, increased connectivity between the DMN and pain and specific migraine-related areas, such as the pons and hypothalamus, developed during the spontaneous migraine attack.

**Conclusion:**

Our preliminary results provide further support for the hypothesis that alterations of the DMN functional connectivity during migraine headache may lead to maladaptive top-down modulation of migraine pain-related areas which might be a specific biomarker for migraine.

## Background

Pain connectome investigations in migraine, using functional magnetic resonance (fMRI) studies during rest and pain suggest that the number of attacks depend on resting-state functional connectivity between the default mode network (DMN), the salience network (SN) and the periaqueductal gray [1, 2]. However, most of these alterations are not migraine-specific and similar biases in functional connectivity can be observed in other chronic pain disorders [3]. In addition, much less is known about the pain connectome signature of acute migraine attacks. Only one study has investigated the effect of pharmacologically induced migraine attack on resting-state functional connectivity, reporting widespread alterations in DMN connectivity with different brain areas [4]. To our knowledge there are no data about spontaneous migraine pain connectome available in the scientific literature so far. In our present work we analysed resting-state fMRI data of a patient with migraine without aura who participated in two fMRI scans and suffered a migraine attack on the second occasion.

## Case presentation

Here we report a case of a 24-year old female migraine without aura patient who participated in two resting-state fMRI sessions (31 days apart) in our study. The diagnosis of migraine was made by a headache specialist according to the International Headache Society criteria [5]. The headache attacks occurred approximately 4 times per month since age 14. They were severe in intensity (7–8 on a scale of 1–10) and had a throbbing quality with nausea, vomiting and photophobia. A typical episode lasted 4–72 h. Her migraine was not related to menstrual cycle. The patient had no other medical conditions and had not taken any daily medications except a contraceptive (gestodene, ethinylestradiol). Both the interictal and ictal scans were carried out on pill-taking days. Migraine attacks were treated with diclofenac and domperidone for nausea.

During the 3 days preceding the fMRI scans she was pain- and medication-free both times. At the second occasion she suffered an unexpected migraine attack during the examination that she subsequently described as a bilateral, throbbing pain (7/10) aggravated by head motion and associated with photophobia and nausea, and after the scan she vomited. In the beginning of the ictal image recording the pain was moderate (3/10) and pulsating with mild photophobia.

Functional MRI data acquisition was performed in the MR Research Center (Semmelweis University, Budapest, Hungary) with a 3 T Achieva MRI scanner (Philips Medical Systems, Best, The Netherlands) using a BOLD-sensitive T2*-weighted echo-planar imaging sequence (TR = 2.500 ms, TE = 30 ms, FOV = 240 × 240 mm^2^) with 3 × 3 mm in-plane resolution and contiguous 3-mm slices providing whole brain coverage. A series of anatomical images were also acquired during the first imaging session using T1-weighted 3D TFE sequence with 1 × 1 × 1 mm resolution. To determine the significant alterations in the functional connectivity, a period of 4 min from both sessions was selected, while the patient was in rest and with eyes opened. Imaging data were analysed using statistical parametrical mapping 12 (SPM 12, Friston, The Welcome Department of Cognitive Neurology, London, UK) and the CONN toolbox for MATLAB, using seed-to-voxel whole brain analysis, with false discovery rate (FDR) correction at p ≤ 0.05 significance level and a minimum cluster size limit of 50 voxels. The DMN seed region was chosen according to the work of Amin and colleagues [4] [posterior cingulate cortex, Montreal Neurological Institute (MNI) coordinates −2 −60 36, R = 10 mm) to make our results comparable to the reported connectivity changes obtained during PACAP38-induced migraine attacks.

The study was approved by the Scientific and Research Ethics Committee of the Medical Research Council, Budapest, Hungary (23609-1/2011-EKU) and was carried out according to the Declaration of Helsinki. The subject gave written informed consent.

## Results

**Fig. 1:**
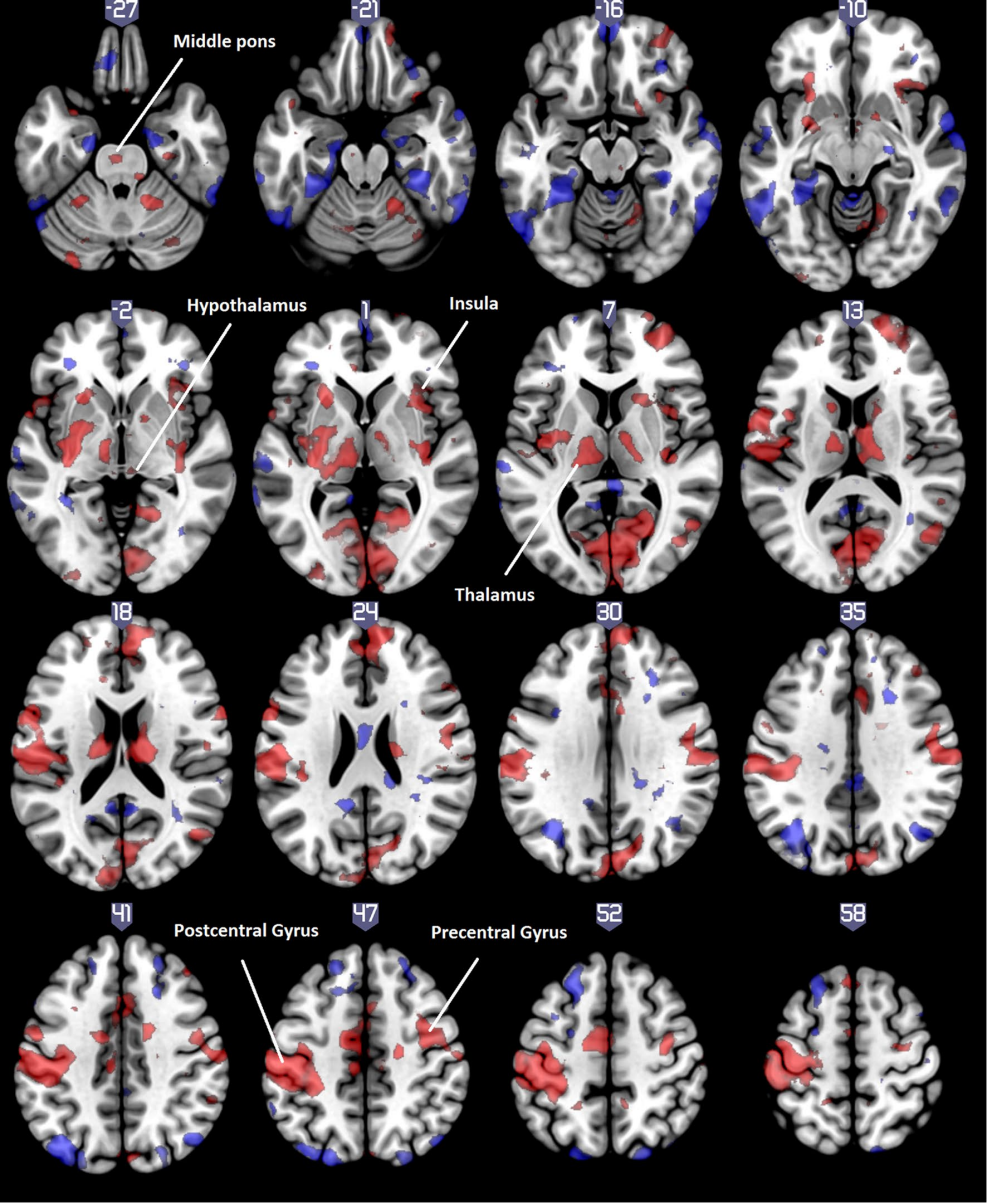
Altered resting-state functional connectivity with DMN during spontaneous migraine attack compared to interictal phase, axial view, pain-related regions written out. *Red*: ictal > interictal, *Blue*: interictal > ictal. (p < 0.05, FDR corrected; no cluster size limit)

During spontaneous migraine attack the DMN showed increased functional connectivity with brain areas related to pain, e.g. thalamus, insula and left postcentral gyrus, and decreased connectivity within the DMN (e.g. precuneus, ventral medial prefrontal cortex, angular gyrus). However, a subsystem of the DMN containing the dorsal medial frontal cortex (dMFC), the superior frontal gyrus (SFG) and the mid temporal gyrus (MidTG) showed increased FC. In addition, increased functional connectivity appeared with the pons and the hypothalamus (Table 1; Fig. 1).

**Table 1:** Altered resting-state functional connectivity with DMN during spontaneous migraine attack compared to interictal phase

Conn	Brain regions	Side	Number of voxels	MNI coordinates			Conn	Brain regions	Side	Number of voxels	MNI coordinates		
				X	Y	Z					X	Y	Z
Increased	Lingual gyrus, cuneus	B	4691	26	−66	4	Decreased	ITG, Fusiform gyrus	L	2125	−56	−72	−14
	IOG	L	156	−36	−100	−8			R	1004	58	−30	−34
	**IPG, PostCG, PreCG, Thal, Caud, hypothalamus**	B	10,486	−46	−24	44		ITG	L	80	−68	−32	−20
	ITG	R	61	52	−18	−36		MidTG	R	516	58	2	−10
	STG	L	55	−30	10	−26		STG	L	384	−66	−24	8
		L	253	−60	10	−2			R	280	40	16	−38
	MidTG	R	423	46	−68	16		Fusiform gyrus	R	467	34	−32	−18
	***dMFC***, SFG	B	1449	10	56	24		Gyrus rectus, ***vMFC***	L	668	−6	40	−24
	SFG	L	86	−20	56	14		MidFG	R	451	22	20	38
	***dMFC***	L	63	−4	28	58			L	129	−34	40	−2
	MidFG	R	289	38	62	−16			L	60	−24	−4	56
		R	87	42	36	40		SFG	L	573	−22	26	54
	**SMA**	B	68	−4	16	64			R	70	8	4	72
	**PreCG, PostCG**	R	1585	36	−10	50		IFG	R	278	34	34	−18
	**PreCG**	L	145	−10	−34	70		**PreCG**	R	79	16	−32	66
	**Insula, claustrum, putamen, pallidum**	R	327	36	−18	4		*Angular gyrus*	R	943	42	−66	36
	MCC	R	189	16	−2	40			L	1403	−38	−68	36
	PHC	R	52	28	−20	−26		**PostCG**	R	84	46	−24	64
	**Pons**	L	52	−4	−22	−26		***PCC, precuneus***	R	438	6	−52	16
	**Putamen**	L	325	−26	16	0			R	101	2	−38	8
	Cerebellum	R	613	28	−56	−60		MCC	R	133	14	−34	24
		L	336	−28	−54	−52			B	127	4	−34	36
		R	177	4	−70	−40		Hippocampus	R	288	22	−16	−6
		L	610	−30	−86	−28		Cerebellum	R	65	10	−54	−52
		L	172	−24	−46	−28			B	182	2	−44	−12
		R	133	36	−72	−24							

Italic part of DMN; bold part of pain matrix

*Conn* connectivity, *R* right, *L* left, *B* bilateral, *dMFC* dorsal medial frontal cortex, *IFG* inferior frontal gyrus, *IOG* inferior occipital gyrus, *IPG* inferior parietal gyrus, *ITG* inferior temporal gyrus, *MCC* middle cingulate gyrus, *MidFG* middle frontal gyrus, *MidTG* middle temporal gyrus, *PCC* posterior cingulate cortex, *PHC* parahippocampal gyrus, *PostCG* postcentral gyrus, *PreCG* precentral gyrus, *SFG* superior frontal gyrus, *SMA* supplementary motor area, *STG* superior temporal gyrus, *Thal* thalamus, *Caud* caudate, *vMFC* ventral medial frontal cortex (p < 0.05, FDR corrected; cluster size limit = 50 voxel)

## Conclusions

Our results demonstrated that during spontaneous migraine attack the intrinsically salient migraine pain decreased functional connectivity within core regions of the DMN, and in parallel connectivity increased between the DMN and SN as a marker of attentional shift towards pain, and also increased between the DMN and regions of pain matrix [2]. These findings support the idea of dynamically changing functional connectivity during migraine cycle, similarly to an earlier longitudinal resting state fMRI study [6].

However, some functional connectivity changes during migraine pain may contribute to longstanding alterations in brain function and thus promote the recurrence of migraine attacks. For example, a subsystem of the DMN, namely the dMFC, SFG and MidTG showed increased functional connectivity with the DMN seed represented by PCC, both in our spontaneous migraine case and during pharmacologically induced migraine attacks [4], which plays an important role in pain interpretation and control [7], and showed a significant difference between migraine without aura patients and controls during interictal phase [8]. In addition, the dMFC which is not only part of the DMN but belongs to the pain connectome, has an important role in pain modulation through shifting attention towards and away from the unpleasant sense [9] and has increased functional connectivity with other DMN regions in chronic pain patients [10, 11]. This altered FC has been found to be positively correlated with pain rumination which is an indicator of unfavourable clinical outcome in chronic pain patients [10].

A previous study reported enhanced functional coupling between the hypothalamus and the middle pons during migraine attack in a patient in response to painful stimuli [12]. In our case, the DMN seed functional connectivity increased with a special region in the pons that was previously identified as migraine generator [13], and with the hypothalamus that is in line with the recent hypothesis which suggested that migraine attacks might be initiated by functional connectivity changes between the hypothalamo–brainstem areas [12].

Despite that the present work is about a single case, and both our and previous studies investigated only female migraine patients [4, 12], these preliminary results further emphasize that alterations of the DMN functional connectivity during migraine attacks may lead to neuroplastic changes in the frontal cortical pain modulating network, which can lead to maladaptive top-down control of the hypothalamo–brainstem areas. Thus resting-state functional connectivity changes in migraineurs might be neuroimaging biomarkers for migraine pathophysiology. In the future, large prospective studies are required to investigate the resting state functional connectivity changes during both spontaneous and pharmacologically induced migraine attacks and to compare them to normal fluctuations in healthy control groups, to verify this hypothesis.
